# Anatomical parameters alter the biomechanical responses of adjacent segments following lumbar fusion surgery: Personalized poroelastic finite element modelling investigations

**DOI:** 10.3389/fbioe.2023.1110752

**Published:** 2023-02-13

**Authors:** Mohammad Nikkhoo, Wen-Chien Chen, Meng-Ling Lu, Chen-Ju Fu, Chi-Chien Niu, Hen-Yu Lien, Chih-Hsiu Cheng

**Affiliations:** ^1^ School of Physical Therapy and Graduate Institute of Rehabilitation Science, College of Medicine, Chang Gung University, Taoyuan, Taiwan; ^2^ Bone and Joint Research Center, Chang Gung Memorial Hospital, Linkou, Taiwan; ^3^ Department of Biomedical Engineering, Science and Research Branch, Islamic Azad University, Tehran, Iran; ^4^ Department of Orthopedic Surgery, Chang Gung Memorial Hospital, Taoyuan, Taiwan; ^5^ Department of Orthopedic Surgery, Chang Gung Memorial Hospital, Kaohsiung, Taiwan; ^6^ Division of Emergency and Critical Care Radiology, Chang Gung Memorial Hospital, Linkou, Taiwan; ^7^ Department of Orthopedic Surgery, Chang Gung Memorial Hospital, Linkou, Taiwan

**Keywords:** personalized modeling, finite element analysis, posterior lumbar fusion, adjacent segment disease, spine biomechanics

## Abstract

**Introduction:** While the short-term post-operative outcome of lumbar fusion is satisfying for most patients, adjacent segment disease (ASD) can be prevalent in long-term clinical observations. It might be valuable to investigate if inherent geometrical differences among patients can significantly alter the biomechanics of adjacent levels post-surgery. This study aimed to utilize a validated geometrically personalized poroelastic finite element (FE) modeling technique to evaluate the alteration of biomechanical response in adjacent segments post-fusion.

**Methods:** Thirty patients were categorized for evaluation in this study into two distinct groups [i.e., 1) non-ASD and 2) ASD patients] based on other long-term clinical follow-up investigations. To evaluate the time-dependent responses of the models subjected to cyclic loading, a daily cyclic loading scenario was applied to the FE models. Different rotational movements in different planes were superimposed using a 10 Nm moment after daily loading to compare the rotational motions with those at the beginning of cyclic loading. The biomechanical responses of the lumbosacral FE spine models in both groups were analyzed and compared before and after daily loading.

**Results:** The achieved comparative errors between the FE results and clinical images were on average below 20% and 25% for pre-op and post-op models, respectively, which confirms the applicability of this predictive algorithm for rough pre-planning estimations. The results showed that the disc height loss and fluid loss were increased for the adjacent discs in post-op models after 16 h of cyclic loading. In addition, significant differences in disc height loss and fluid loss were observed between the patients who were in the non-ASD and ASD groups. Similarly, the increased stress and fiber strain in the annulus fibrosus (AF) was higher in the adjacent level of post-op models. However, the calculated stress and fiber strain values were significantly higher for patients with ASD.

**Discussion:** Evaluating the biomechanical response of pre-op and post-op modeling in the non-ASD and ASD groups showed that the inherent geometric differences among patients cause significant variations in the estimated mechanical response. In conclusion, the results of the current study highlighted the effect of geometrical parameters (which may refer to the anatomical conditions or the induced modifications regarding surgical techniques) on time-dependent responses of lumbar spine biomechanics.

## 1 Introduction

The posterior instrumentation with rigid-rod fusion is considered the gold standard surgical treatment of pathologies such as lumbar instability, spinal stenosis, spondylolysis, and spondylolytic spondylolisthesis (Serhan et al., 2011; Kim and Choi, 2018). Although the short-term post-operative (post-op) outcome of lumbar fusion is satisfying for most patients, adjacent segment disease (ASD) can be prevalent in long-term clinical observations (Kim et al., 2016). This long-term phenomenon mainly affects the adjacent intervertebral discs (IVDs), however, it can reveal instability, retro-spondylolisthesis, and fracture in adjacent vertebrae, as well (Liang et al., 2014; Kim et al., 2016).

Different risk factors have been proposed for increasing the chance of ASD development in patients [such as age, sex, body mass index (BMI), and osteoporosis], non-etheless, the post-op ASD alteration may possibly be a result of induced modifications in lumbosacral spine lordosis angle, kinematics, and kinetics (Helgeson et al., 2013; Vergroesen et al., 2015; Ebrahimkhani et al., 2022). Hence, characterizing the effect of lumbar fusion on biomechanical responses of adjacent levels could be beneficial for surgeons towards improved surgical planning and enhanced clinical outcomes. While various *in-vitro* experimental (Phillips et al., 2006; Erbulut et al., 2013; Beckmann et al., 2020) and clinical investigations (Kim et al., 2012; Hsieh et al., 2016; Zhang et al., 2018a) have been performed to compare the outcome of using different posterior fusion devices for treatment of the lumbar spine diseases, there is no access to a non-invasive technique to evaluate the post-op alterations in the spinal biomechanics. Finite element (FE) analysis could be similarly employed as a conventional predictive approach for clinical investigations due to its ability to represent the complex systems and predict their response (Dreischarf et al., 2014).

Despite the proven success of FE analyses for investigation of the lumbar spine post surgeries (Zhang et al., 2018b; Zhang et al., 2018c; Zhao et al., 2018), their application in clinical investigations, as an assistive tool, may be questioned by clinicians. The differences in the spine anatomical geometry may possibly cause uncertainty in FE model outputs and limit the reliability of achieved predictions (Laville et al., 2009). Geometrically personalized FE modeling, regardless of its simplifications, can provide a modular tool for clinical studies that include patient-specific characterization using clinical images to account for the variability between different patients (Nikkhoo et al., 2020; Ebrahimkhani et al., 2022).

Hence, to fill the gap of knowledge, it might be valuable to investigate if inherent geometric differences among patients can significantly alter the biomechanics of adjacent levels post-surgery. We recently developed a geometrical personalized FE modeling technique in which the detailed time-dependent fluid-solid interactive response was considered to enhance prediction under both static and dynamic loading conditions (Nikkhoo et al., 2021). The main objective of this study was to utilize this modeling technique to evaluate if the geometrically personalized FE modeling can predict the alterations in lumbar adjacent levels post-fusion surgery. For this purpose, a prospective, non-randomized cohort study was performed in which the patients underwent one-level lumbar interbody fusion and it is hypothesized that the FE modeling technique may identify remarkable variations in adjacent segment kinematics and kinetics between patients without ASD and patients with ASD.

## 2 Materials and methods

### 2.1 Geometrical personalized FE modeling of the pre-operative lumbosacral spine

**FIGURE 1:**
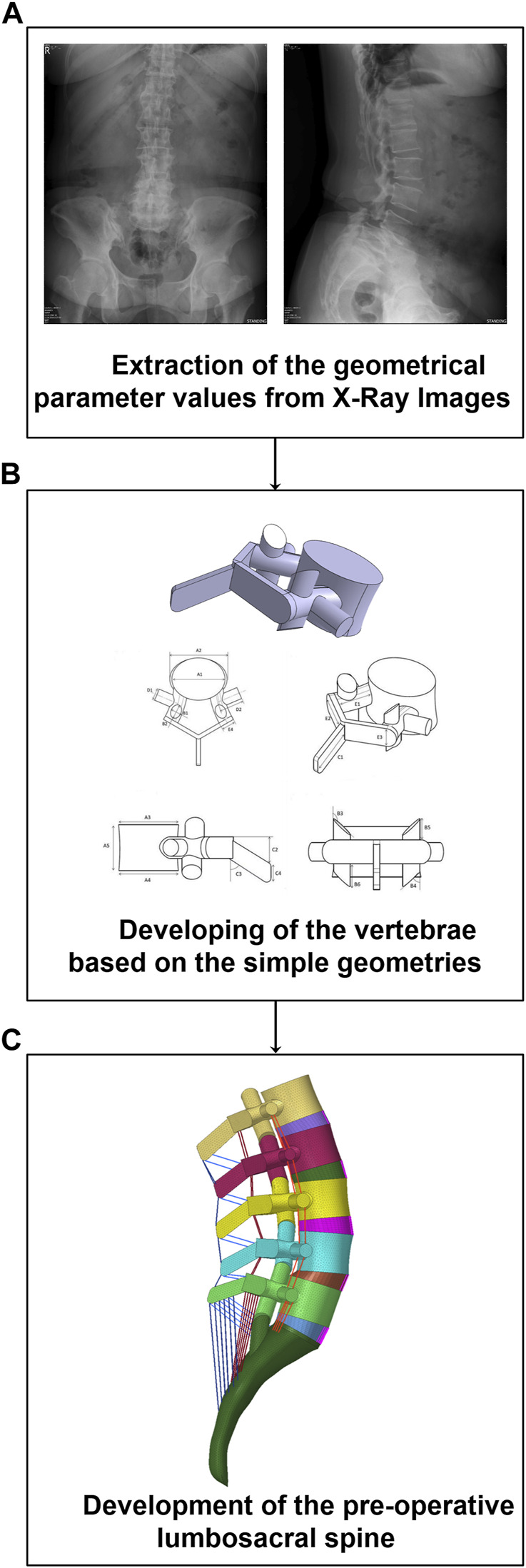
Methodology of personalized pre-operative finite element (FE) modeling of the lumbosacral spine form from lateral and anterior posterior (AP) X-ray radiographs. **(A)** Parameters extraction, **(B)** Development of the vertebrae, **(C)** Development of the FE model.

The pre-operative geometries of the lumbosacral spine (L1-S1) of 30 patients were generated from lateral and anterior-posterior (AP) X-ray radiographs (Age: 64.8 ± 8.1 years, BMI: 26.2 ± 3.5 kg/m^2^, 25 females and 5 males) using a previously developed validated geometrical modeling procedure (Nikkhoo et al., 2020) (Figure 1). The X-ray radiographs were selected from a prospective, non-randomized cohort study in which the patients underwent one-level lumbar interbody fusion at our hospital from 2008 to 2018. The pathologies for surgery were disc degeneration disease, spondylolisthesis, and segmental instability in the lumbar region and none of the selected patients had a history of previous spinal surgery. Based on a long-term follow-up study, the patients were divided into two groups [i.e., 1) ASD group (N = 15) and Non-ASD group (N = 15)]. The patients in the ASD and non-ASD groups were classified based on clinical and radiological indices after long-term follow-up investigations (5.37 ± 3.18 years). This study was approved by Chang Gung Memorial Hospital’s research ethics committee (approval No. 201702031B0) and signed informed consent was acquired from all participants prior to their enrolment in the relevant clinical protocol.

A non-linear poro-hyperelastic FE model of the lumbosacral spine (L1-S1) was developed for each patient based on their extracted geometrical values from pre-operative (pre-op) X-ray images (Figure 1). Each FE model consists of bony parts (i.e., posterior bony elements and vertebral bodies including cancellous and cortical bones) and soft tissues (i.e., five IVDs and ten cartilaginous endplates, seven ligaments, and five pairs of cartilaginous facet joints). The IVDs were characterized in FE modeling by a reinforced complex material consisting of the annulus fibrosus (AF) ground matrix reinforced with AF collagen fibers, and nucleus pulposus (NP). The drained solid phase of bony parts was considered isotropic elastic. However, the drained solid phase of the AF matrix and NP region were replicated based on the non-linear Mooney–Rivlin hyperelastic elastic-plastic hardening theory based on relevant studies in the literature (Schmidt et al., 2007; El-Rich et al., 2009). In addition, the theory of poroelastic (Argoubi and Shirazi-Adl, 1996; Ferguson et al., 2004) was reflected in the time-dependent response of the bony parts, cartilaginous endplates, and IVDs in the FE model. For this purpose, the values of permeability were reflected by variables based on the calculated void ratio in simulations based on the following equation (Argoubi and Shirazi-Adl, 1996; Ferguson et al., 2004),

$$k=k_{0}{\left[\frac{e\left(1+e_{0}\right)}{e_{0}\left(1+e\right)}\right]}^{2}\exp\left[M\left(\frac{1+e}{1+e_{0}}-1\right)\right]$$ (1)

Where *k*_*0*_ is the input initial permeability and *e* is defined as follows,

$$e=\frac{\emptyset_{f}}{1-\emptyset_{f}}$$ (2)

Where Ø_f_ is the porosity of the tissue which varies with material deformation during FE calculations. The composite structure of AF was mimicked by embedding six concentric reinforced fiber lamellae with an orientation of ±35° within a distance of 1 mm in the AF ground substance (Naserkhaki et al., 2016a). A constant boundary pore pressure equal to 0.25 MPa was imposed on all external surfaces of the IVDs as an additional constraint to include the swelling sensation in IVDs (Schmidt et al., 2010; Galbusera et al., 2011a). Ligaments were represented in the FE model using non-linear truss elements which were connected to bony parts and their length could be updated based on the patient’s geometrical input values. The mechanical properties of the ligaments were non-linear elastic based on available data in the literature and they could only be activated in tension (Shirazi-Adl et al., 1986a; Pintar et al., 1992). The mechanical properties of different components in this lumbosacral spine FE model are presented in Table 1.

**TABLE 1:** Mechanical properties of the geometrical personalized poroelastic finite element model.

Component	Mechanical property behavior	Values of the mechanical properties	References
Cortical bone	Linear poroelastic	E = 12,000 MPa, ν = 0.3, *k*_*0*_ = 1 × 10^−20^ (m^4^/Ns), e = 0.02	Argoubi and Shirazi-Adl (1996), Goto et al. (2003), Ferguson et al. (2004), Schmidt et al. (2010), Galbusera et al. (2011b), Park et al. (2013)
Cancellous bone	Linear poroelastic	E = 200 MPa, ν = 0.25, *k*_*0*_ = 1 × 10^−13^ (m^4^/Ns), e = 0.4	Argoubi and Shirazi-Adl (1996), Ferguson et al. (2004), Schmidt et al. (2007), Schmidt et al. (2010), Galbusera et al. (2011b), Shih et al. (2013)
Endplate	Linear poroelastic	E = 5 MPa, ν = 0.1, *k*_*0*_ = 7.5 × 10^−15^ (m^4^/Ns), e = 4	Argoubi and Shirazi-Adl (1996), Goto et al. (2003), Ferguson et al. (2004), Schmidt et al. (2007), Schmidt et al. (2010), Galbusera et al. (2011b)
Annulus fibrosus matrix	Incompressible Poro-Hyperelastic (Mooney-Rivilin)	C10 = 0.18, C01 = 0.045, *k*_*0*_ = 3 × 10^−16^ (m^4^/Ns), e = 2.33	Argoubi and Shirazi-Adl (1996), Ferguson et al. (2004), El-Rich et al. (2009), Schmidt et al. (2010), Galbusera et al. (2011b)
Nucleus pulposus	Incompressible Poro-Hyperelastic (Mooney-Rivilin)	C10 = 0.12, C01 = 0.030, *k*_*0*_ = 7.5 × 10^−16^ (m^4^/Ns), e = 4	Argoubi and Shirazi-Adl (1996), Ferguson et al. (2004), Schmidt et al. (2007), Schmidt et al. (2010), Galbusera et al. (2011b)
Collagen fibers	Non-linear elastic	The fiber stiffness increases from the inner to the outer layer	Shirazi Adl et al. (1986b), Schmidt et al. (2006)
*ALL, PLL, LF, ISL, SSL, ITL, CL	Non-linear elastic	Non-linear curves from the literature	Shirazi-Adl et al. (1986a), Pintar et al. (1992)
Pedicle screws	Elastic	E = 110,000 MPa, ν = 0.3	Zhang et al. (2018c)
Rigid rod (Ti)	Elastic	E = 110,000 MPa, ν = 0.3	Zhang et al. (2018c)
Interbody PLIF cage	Elastic	E = 3,500 MPa, ν = 0.3	Zhang et al. (2018c)

*ALL, anterior longitudinal ligament; PLL, posterior longitudinal ligament; LF, ligamentum flavum; ISL, interspinous ligament; SSL, supraspinous ligament; ITL, intertransverse ligament; CL, capsular ligament.

The vertebrae, cartilaginous endplates, and IVDs in each level were attached together in their anatomical positions using surface-to-surface tie contact conditions which provide equal translational and rotational motions at connected nodes. The facet joint surfaces were approximated in the model by a plane in which its orientation was defined by two card angles (Van Schaik et al., 1985; Panjabi et al., 1993). Based on the previous data in the literature (Panjabi et al., 1993) and our sensitivity analyses, the card angle about the x-axis was considered constant (80 degrees) and the one about the y-axis was chosen as the variable parameter which is extracted from the AP image. A surface-to-surface contact algorithm for both normal and tangential directions was considered to represent the articulation of the facet joints. A soft frictionless contact within an initial gap length of 0.5 mm to mimic the articulation of the facet joints in the FE model was considered (Naserkhaki et al., 2016a; Naserkhaki et al., 2016b; Naserkhaki and El-Rich, 2017). The transmitted force through contacting surfaces was mimicked using an exponential pressure-overclosure from zero at the initial gap until the contact pressure reached 120 MPa (Schmidt et al., 2010; Naserkhaki et al., 2016a; Naserkhaki et al., 2016b). To optimize the spine stability under compression while it lacks muscles, the weight of the upper body was applied as a compressive load using the follower load technique in which the line of action followed the spine curvature and passed through the vertebral bodies’ centroids (Patwardhan et al., 1999; Shirazi-Adl and Parnianpour, 2000; Dreischarf et al., 2014; Naserkhaki et al., 2016a; Naserkhaki and El-Rich, 2017). This follower load was applied to the models using pre-compressed unidirectional springs inserted between the centroids of two adjacent vertebral bodies. The rotational moments in different directions (i.e., flexion, extension, left/right lateral bending, and left/right axial rotation) were applied to the superior surface of the lumbosacral spine (L1) and Dirichlet boundary settings were applied at the sacral region to inhibit any displacement/rotation in all degrees of freedom. The model verification was approved based on mesh sensitivity analyses, and the finalized FE models contain 186,325 elements.

The overall validity of this FE modeling was previously confirmed for both static (Nikkhoo et al., 2020) and cyclic loading (Nikkhoo et al., 2015; Khalaf and Nikkhoo, 2021) which was well-aligned with both numerical and experimental studies (Rohlmann et al., 2009; Dreischarf et al., 2011; Dreischarf et al., 2012; Dreischarf et al., 2014). To evaluate the usability of the developed FE modeling technique in clinical applications, both pre- and post-operation functional X-ray images (patient in neutral, flexion, and extension positions) were employed to validate the predicted intersegmental range of motion (ROM). The functional X-ray images were used to measure the total lordosis angles (L1-S1) in neutral, flexion, and extension positions, while the ROM for each patient during flexion and extension was measured. The calculated subject-specific rotation of the L1 vertebra was then applied to the FE model, and the predicted rotations of each vertebra (i.e., intersegmental ROM) were compared with the measured ones from the images. To minimize the simplification errors regarding the consistency of the boundary conditions of the model with the *in-vivo* lumbar spine, a rotational control technique was utilized in the validation phase. To compare the achieved results, the percentage of the root mean square error (RMSE) was calculated as follows,

$$RMSE=\sqrt{\frac{\overset{i=N}{\underset{i=1}{\sum }}{\left(\frac{{\left({ROM}_{X-Ray\;Image}\right)}_{i}-{\left({ROM}_{FEM}\right)}_{i}}{{\left({ROM}_{X-Ray\;Image}\right)}_{i}}\right)}^{2}}{N}}$$ (3)

where, “N” was considered equal to 5, corresponding to 5 spinal levels (L1-L2, to L5-S1) for the pre-op FE model.

### 2.2 Geometrical personalized FE modeling of the post-operative lumbosacral spine

To evaluate if the developed geometrical personalized FE models can predict any differences between biomechanical responses of the non-ASD group versus the ASD patient group, the post-operative (post-op) models of each patient were developed based on the X-ray images which were obtained 3 months post-surgery for both groups. To mimic the fusion surgery, a wide laminectomy was simulated by removing the relevant bony parts, IVD, posterior longitudinal ligament (PLL), and ligamentum flavum (LF). A posterior bilateral pedicle screw fixation system (including four pedicle screws, and two Titanium rods) was implanted and two posterior lumbar interbody fusion (PLIF) cages were inserted in the fusion level. The geometrical parameters of the fusion level (i.e., pedicle screw size, interbody cage height, and lordosis angle) were carefully adapted based on the post-op X-ray images. The material properties of the screws, rods, and interbody PLIF cages were considered isotropic elastic from literature (Zhang et al., 2018c) (Table 1). The tie contact boundary condition was set to constrain equal displacement during calculations (i.e., the same rotational and translational movement) for connected surfaces between the vertebrae, screws, and rods for mimicking the permanent fusion. In addition, the rod surfaces were directly connected to the screws using a tie contact technique to mimic fastening the screws using nuts in the fixation system.

**FIGURE 2:**
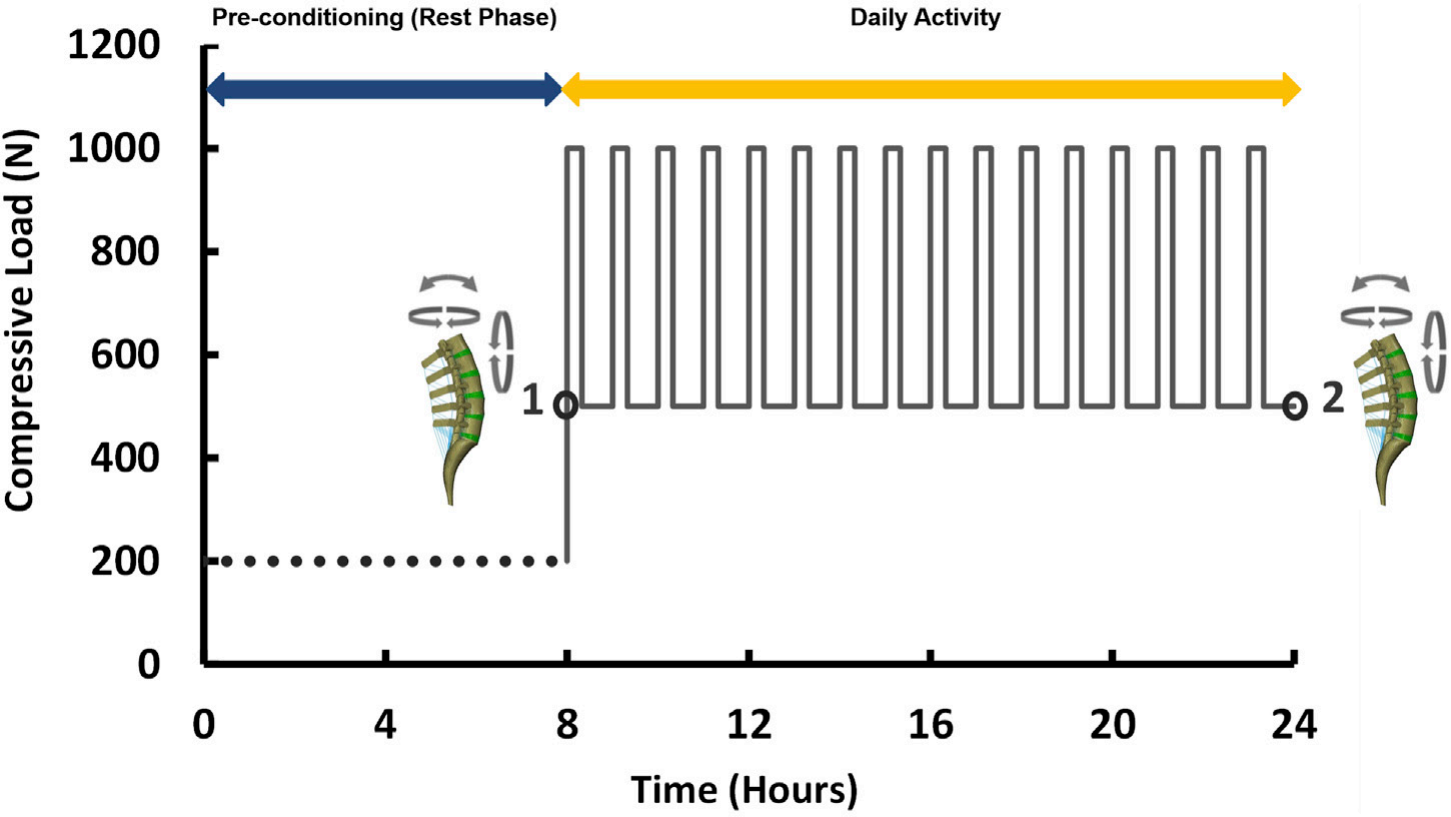
Loading scenario of the daily cyclic loading (Flexion, extension, lateral bending, and axial rotation moments of 10 N m were applied at points 1 and 2).

Similar to the pre-op FE models, the accuracy of the predictions from post-op FE models were compared with those measured from functional post-op X-ray images during the movement in the sagittal plane. Incidentally, we removed the fused level from RMSE calculations for the post-op models, as this level was fixed during rotation, hence “N” was considered equal to four in Eq. 3. The post-op models for both the ASD and non-ASD groups were simulated under combined loading (i.e., compressive load and 10 Nm rotation in different planes) (Dreischarf et al., 2014) under the same aforementioned boundary conditions. To evaluate the time-dependent responses of the models subjected to cyclic loading, a daily cyclic loading scenario was applied to the FE models [i.e., 16 h of cyclic compressive loading of 500–1,000 N (40 and 20 min, respectively) after an 8 h pre-conditioning resting phase of 200 N (Nikkhoo et al., 2021)]. Different rotational movements (i.e., flexion, extension, right and left lateral bending, right and left axial rotation) were superimposed using a 10 N m moment before and after cyclic loading (i.e., points 1 and 2 in Figure 2) to model the rotational motions in the morning and the evening. The rotational moments were linearly applied and removed after 10s and only one motion was evaluated in each diurnal loading simulation. The biomechanical responses of the lumbosacral FE spine models in both groups were analyzed and compared before and after daily loading.

### 2.3 Statistical analyses for comparison of the results for ASD versus Non-ASD patients

The results of FE models (including the ROM, IVD height loss, intradiscal fluid loss, stress in AF matrix, and AF collagen fiber strain) in ASD and Non-ASD patients were compared. We used the non-parametric Friedman comparative test to specify the differences of the predicted results for ASD versus Non-ASD patients. The *p*-values less than 0.05 were reflected as significant statistical differences in this study.

## 3 Results

**FIGURE 3:**
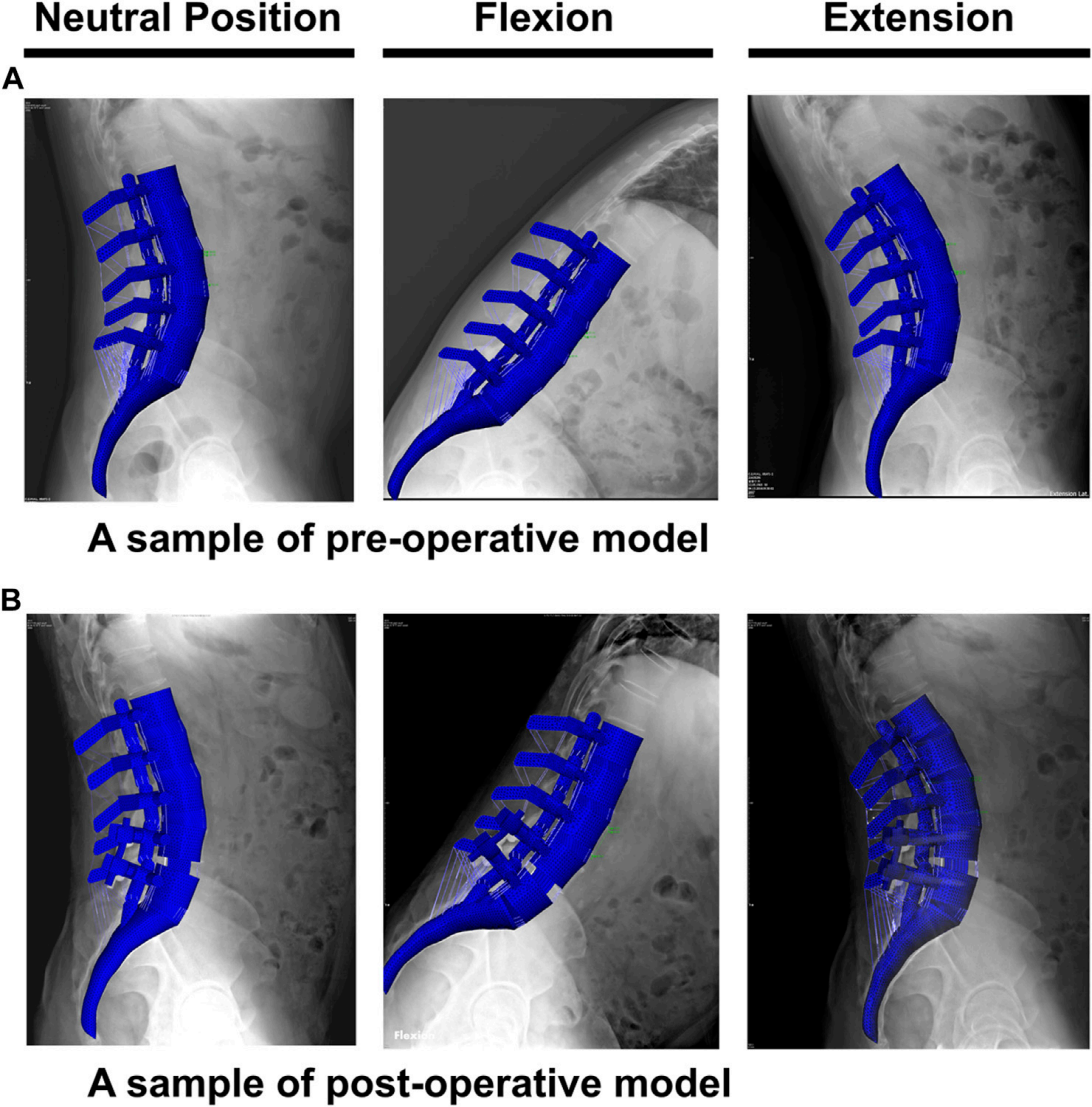
A schematic sample of comparison between the finite element results and the functional X-ray images in the neutral position, flexion, and extension for **(A)** pre-operative and **(B)** post-operative models.

The geometrical personalized pre-op and post-op lumbosacral models were successfully developed for all thirty patients in both ASD and Non-ASD groups using our modeling updating algorithm and the relevant mesh sensitivity analyses confirmed the verification of their predictions. The percentage of calculated RMSE for pre-op FE models were 14.85% and 16.19% in flexion and 17.93% and 17.27% in extension for non-ASD and ASD patients’ models, respectively (Table 2). Correspondingly, these values for post-op models were 23.33% and 20.87% in flexion and 26.23% and 27.64% in extension for non-ASD and ASD patients, respectively (Table 3). Figure 3 schematically presents a sample of FE results overlapped with X-ray images for a patient from the non-ASD group.

**TABLE 2:** Calculated percentage of root mean square errors (RMSE) for evaluating the accuracy and validity of the personalized pre-operative (pre-op) FE models for non-ASD patients (N = 15) and ASD patients (N = 15).

FE model	Percentage of the RMSE for measured and calculated ROM			
	Non-ASD group		ASD group	
	Flexion (%)	Extension (%)	Flexion (%)	Extension (%)
Patient No. 1	13.58	16.32	18.04	15.36
Patient No. 2	11.29	13.39	8.51	14.59
Patient No. 3	21.08	26.49	25.78	28.91
Patient No. 4	8.49	7.29	11.30	12.24
Patient No. 5	24.03	25.30	15.73	23.68
Patient No. 6	17.89	21.37	18.08	25.63
Patient No. 7	11.03	13.58	15.61	19.34
Patient No. 8	14.31	13.78	9.08	8.72
Patient No. 9	24.78	27.50	34.23	27.59
Patient No. 10	6.25	11.33	20.54	26.09
Patient No. 11	16.02	24.11	7.83	16.49
Patient No. 12	12.32	15.98	16.29	13.38
Patient No. 13	7.43	12.63	13.19	18.06
Patient No. 14	15.16	21.22	10.24	14.41
Patient No. 15	19.01	18.59	18.35	24.58
Average	14.85%	17.93%	16.19%	19.27%

**TABLE 3:** Calculated percentage of root mean square errors (RMSE) for evaluating the accuracy and validity of the personalized post-operative (post-op) FE modeling technique.

FE model	Percentage of the RMSE for measured and calculated ROM			
	Non-ASD group		ASD group	
	Flexion (%)	Extension (%)	Flexion (%)	Extension (%)
Model No. 1	16.39	15.78	23.47	30.32
Model No. 2	17.84	22.07	12.49	25.28
Model No. 3	15.43	20.57	34.68	44.30
Model No. 4	12.48	17.42	19.66	23.79
Model No. 5	26.70	35.23	22.08	29.48
Model No. 6	18.48	19.54	15.41	24.54
Model No. 7	16.85	23.93	18.83	24.69
Model No. 8	22.73	28.02	8.40	21.18
Model No. 9	37.01	41.04	38.24	46.32
Model No. 10	9.20	19.07	28.74	33.75
Model No. 11	26.39	38.56	12.55	13.43
Model No. 12	19.69	24.23	21.35	30.61
Model No. 13	13.52	20.63	18.45	18.29
Model No. 14	38.67	36.38	17.83	21.04
Model No. 15	28.57	31.03	24.00	27.59
Average	23.33%	26.23%	20.87%	27.64%

**FIGURE 4:**
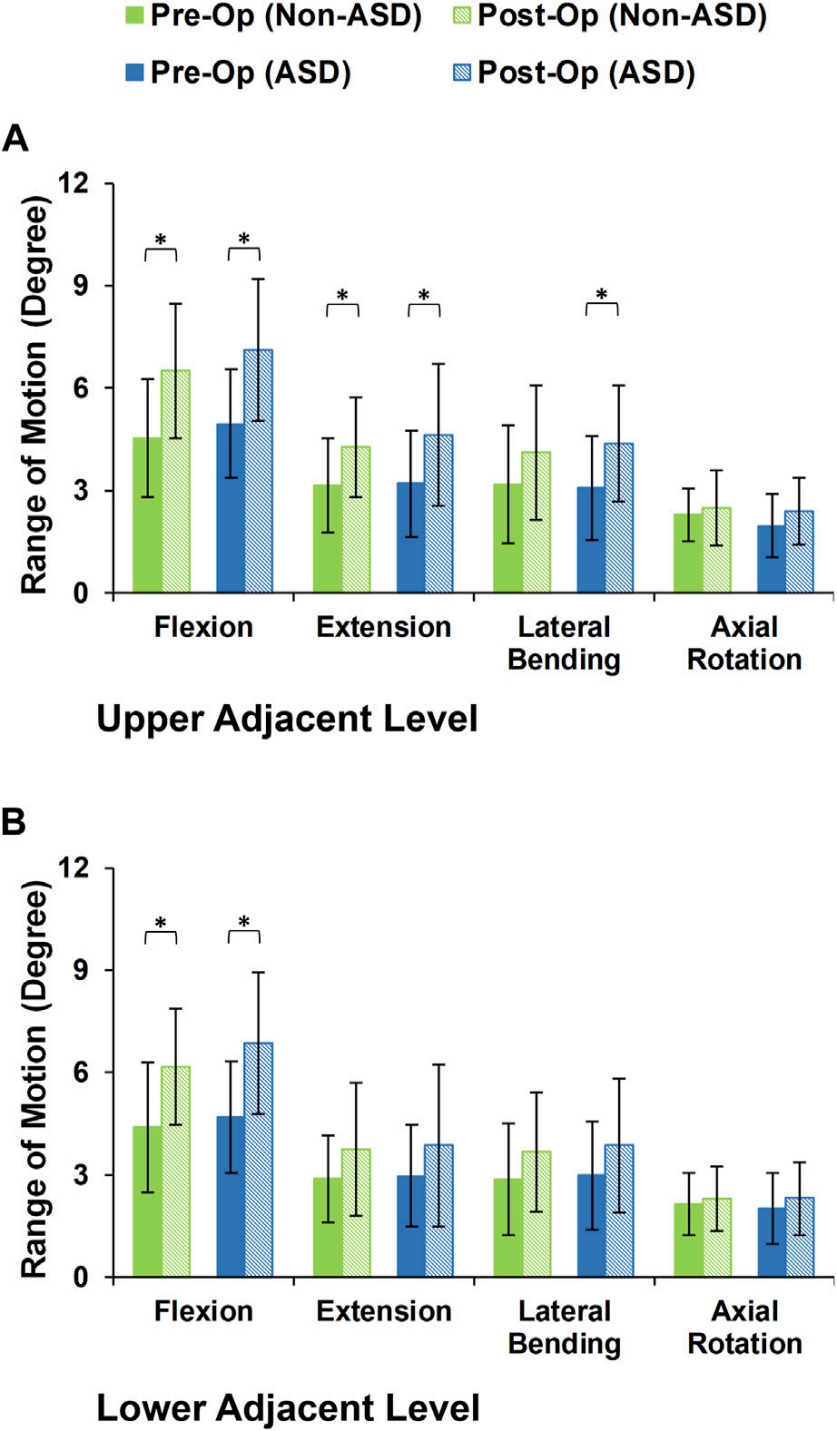
Intersegmental motion patterns for non-ASD and ASD group FE models in the **(A)** upper and **(B)** lower adjacent level for different directions.

**FIGURE 5:**
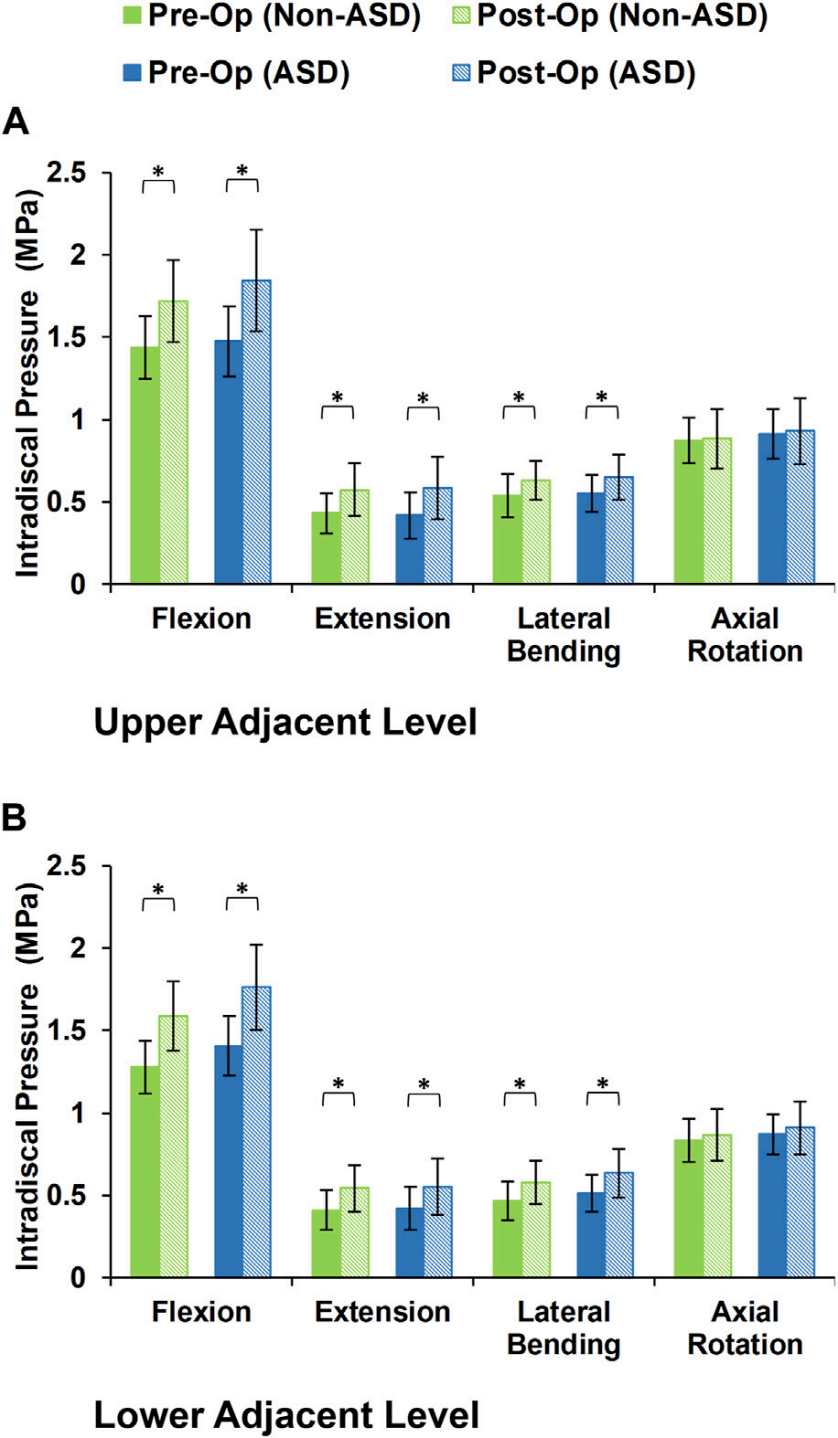
Calculated intradiscal pressure for non-ASD and ASD group FE models in the **(A)** upper and **(B)** lower adjacent level for different directions.

**FIGURE 6:**
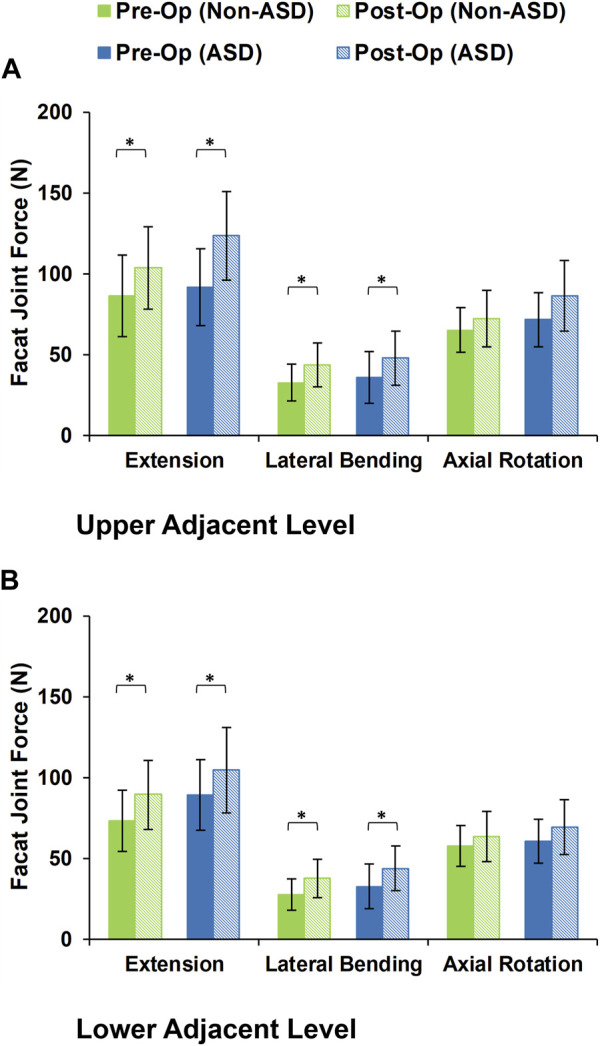
Calculated facet joint forces for non-ASD and ASD group FE models in the **(A)** upper and **(B)** lower adjacent level for different directions.

During the static loading scenario, the average ROM at the upper and lower adjacent levels increased post-surgery for both the non-ASD and ASD groups (Figure 4). The differences in average ROMs between pre-op and post-op results were significant for flexion and extension movements for both groups. However, there was no significant difference when we compared the values between the two groups. The significantly higher ROM in lateral bending was only observed in the ASD group for the upper adjacent level (Figure 4). The calculated values for IDP were quite similar in that significant differences were detected for flexion, extension, and lateral bending in both upper and lower adjacent levels (Figure 5). The FJF values showed a significant increase for extension and lateral bending as well (Figure 6).

**FIGURE 7:**
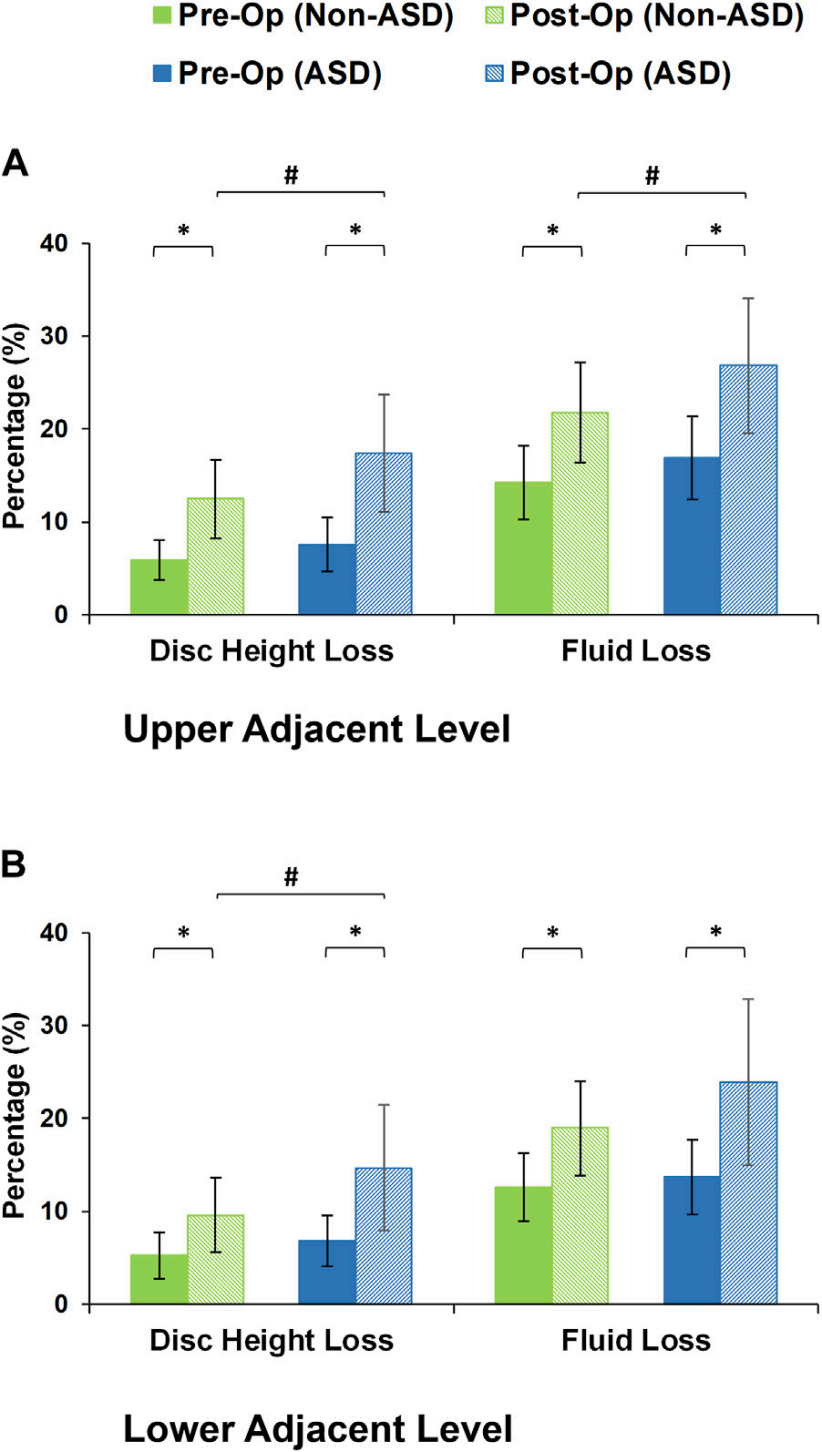
Disc height loss and fluid loss for post-operative non-ASD and ASD group FE models for the **(A)** upper and **(B)** lower adjacent levels.

After daily cyclic loading (i.e., 16 h of cyclic loading to simulate the daily activities), the adjacent disc height averagely decreased by 5.56% and 7.17% in the pre-op models of non-ASD and ASD patients, respectively. These values were 11.03% and 16.03% for the post-op models in the non-ASD and ASD groups, respectively. Significant differences were observed between pre-op and post-op models in both groups (Figure 7). In addition, the differences between the post-op models in the non-ASD and ASD groups were significant for both upper and lower adjacent levels (*p* values equal to 0.016 and 0.021, respectively) (Figure 7). The fluid loss values were on average 13.44% and 15.29% for the pre-op models and 20.38% and 25.36% for the post-op models in the two groups after daily cyclic loading, respectively. The comparative difference between the post-op models in the non-ASD and ASD groups was significant (*p*-value = 0.039) for the upper adjacent level (Figure 7).

**FIGURE 8:**
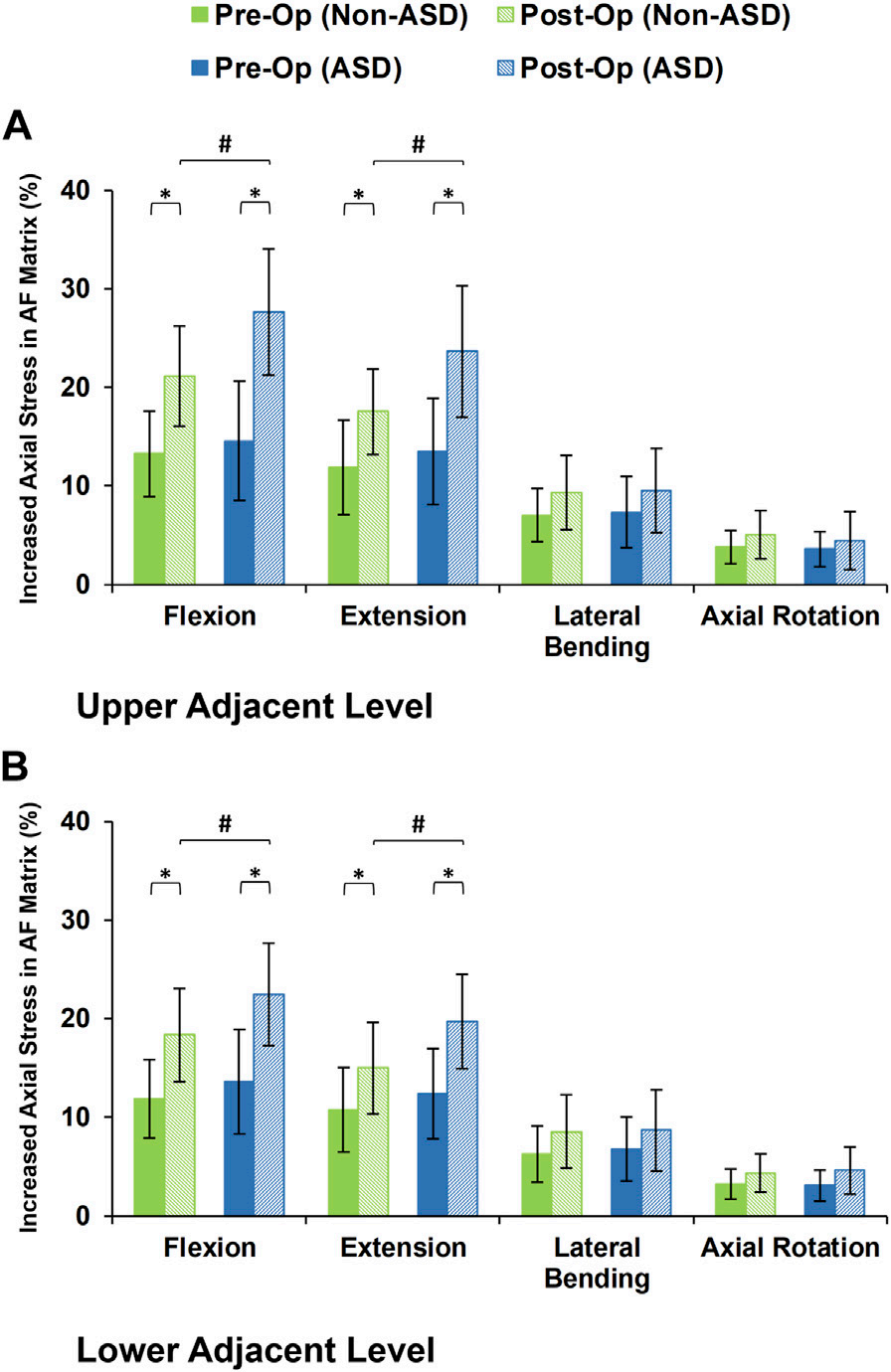
Increased axial stress in AF matrix for post-operative non-ASD and ASD group FE models for the **(A)** upper and **(B)** lower adjacent levels.

**FIGURE 9:**
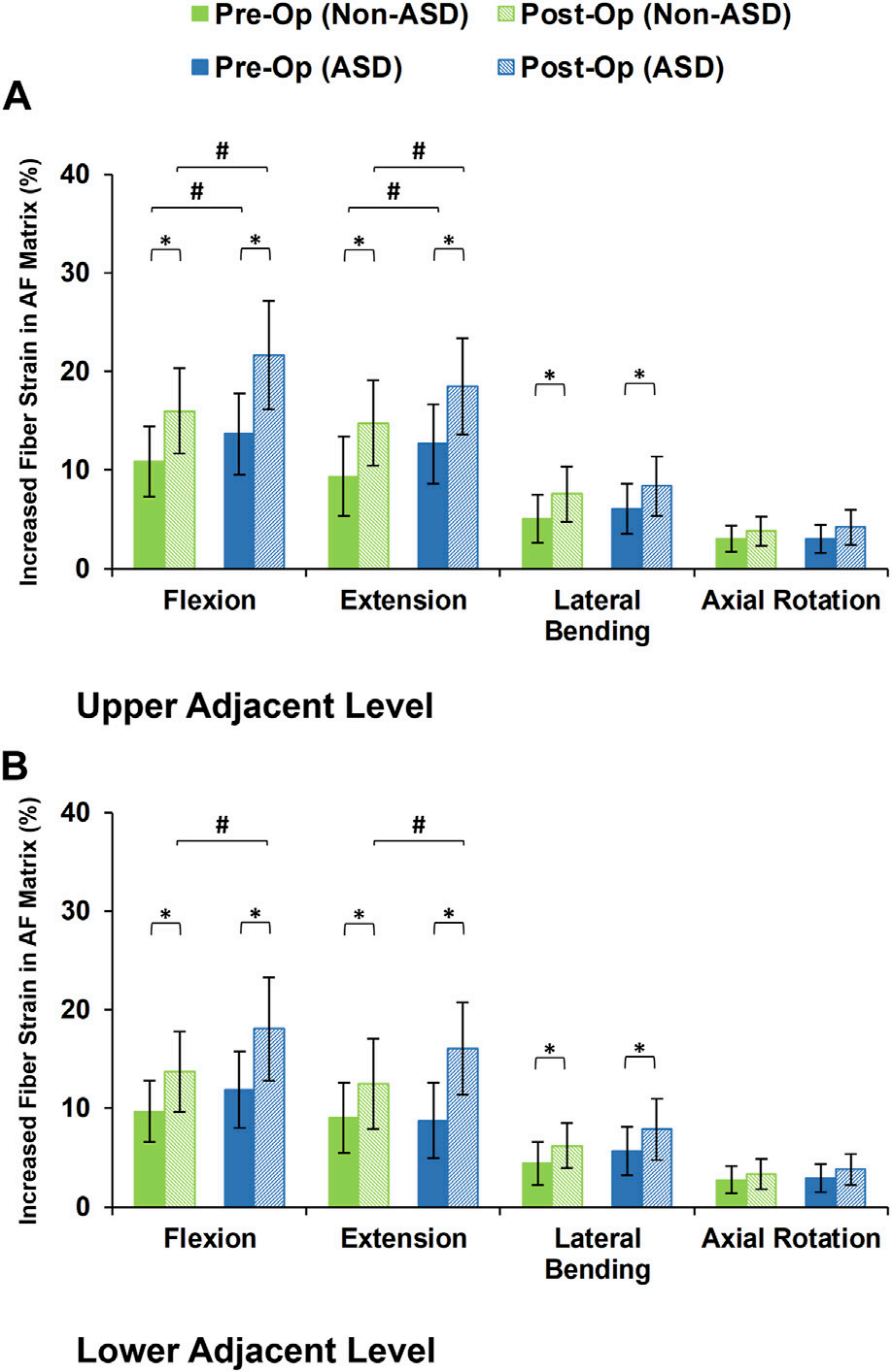
Increased collagen fiber strain in AF matrix for post-operative non-ASD and ASD group FE models for the **(A)** upper and **(B)** lower adjacent levels.

The axial stress in the AF matrix significantly increased after fusion in sagittal plane movement (i.e., flexion and extension) and the differences between the non-ASD and ASD groups were significant in this plane only for the post-op models (Figure 8). However, fusion surgery did not significantly alter the AF axial stress in either lateral bending or axial rotation (Figure 8). Similar trends were calculated for collagen fiber strains. However, the differences between the non-ASD and ASD groups were significant for flexion and extension for both the pre-op and post-op results (Figure 9).

## 4 Discussion

The rigid instrumented posterolateral fusion (PLF) and posterior lumbar interbody fusion (PLIF) techniques are commonly considered the gold standard surgical treatment for different degenerative lumbar pathologies. Understanding lumbar spine biomechanics in post-surgical maneuvers could be beneficial for pre-planning the treatments based on primary rough estimations using personalized FE calculations. Hence, this study utilized a validated geometrically patient-specific FE modeling technique which can be used as a simple and cost-effective tool to evaluate the alteration of biomechanical response in adjacent segments post-fusion. Thirty patients were categorized for evaluation in this study in two distinct groups [i.e., (Serhan et al., 2011) non-ASD (N = 15) and (Kim and Choi, 2018) ASD patients (N = 15)] based on long-term clinical follow-up investigations. Although the current personalized FE modeling technique was previously validated against experimental *in-vitro* and numerical studies, the accuracy of each FE model for these 30 patients was evaluated based on the pre-op and post-op functional X-ray images in the sagittal plane. The achieved errors were averagely below 20% and 25% for pre-op and post-op models which confirms the applicability of this predictive algorithm. The observed error can be tolerated as this technique may be used as a rough estimation for clinicians. Hence, we developed and simulated pre-op and post-op FE models for each patient (in total, 60 FE models were simulated in this study) and evaluated the variations in biomechanical responses of adjacent segments post-surgery. We used the post-op X-ray images after 3 months in which no signs of ASD were observed to investigate if the results of FE simulations could show any significant difference between the non-ASD and ASD groups. Repeating the simulations based on acceptable numbers of patients (which was 15 patients in each group) included the influences of the patient’s anatomical parameters (such as disc height, shape and geometry of vertebra, lumbar lordotic angle before and after surgery, interbody cage height, etc.) to evaluate the aforementioned hypothesis of this study.

ASD may possibly be initiated and developed in 2–5 years based on induced modifications post-surgery, however, it is not possible to simulate this long-term phenomenon using numerical modeling. Meanwhile, most of the available FE studies which investigated the outcomes of different fixation surgical techniques only used static loading and evaluated the model response under simplified loading conditions (Jin et al., 2012; Jahng et al., 2013; Guo et al., 2019; Guo and Yin, 2019). Considering the time-dependent behavior of both vertebra and IVD in lumbar spine motion segments can enhance the accuracy of the biomechanical predictions. One of our contributions to the literature in this study was to utilize the non-linear poroelasticity theory in FE simulations to represent the interaction of interstitial water in a saturated solid matrix which makes the calculations more complicated and laborious. Hence, we provided more realistic simulations to evaluate the biomechanical responses of the lumbosacral spine under a daily cyclic loading regime (i.e., 8 h of resting time prior to 16 h of daily activity) using the aforementioned time-dependent constitutive equations. Using the poroelasticity theory, the interstitial water flow was calculated during daily cyclic loading to extract the variations in disc height and fluid loss. Although a daily cyclic loading (24 h) cannot represent a long-term loading condition, significant variations in output parameters (such as disc height, disc fluid loss, stress in the AF matrix, and strain in collagen fibers) may be characterized as indicators to reflect the initiation of abnormality in the biomechanical response which could be accumulated by repetitive loading.

The primary findings of this study demonstrated that the motion patterns in adjacent segments altered in post-op models. Although a slightly increased in ROM was calculated in most of the post-op FE models in different movement directions, the variation of ROM was significant in the sagittal plane (i.e., flexion and extension). However, no significant difference was observed between the calculated ROM of adjacent segments for the non-ASD and ASD groups. Posterior fusion results in increasing the spinal segmental rigidity which could possibly result in rotational compensation to the adjacent segments (Chiang et al., 2006; Sudo et al., 2006). Based on this hypothesis, the IDP and FJF values were significantly increased in adjacent levels in extension and lateral bending movements as well. Similar to ROM, this static loading condition did not demonstrate any significant difference between the non-ASD and ASD patients.

Simulating the biomechanical response of the pre-op and post-op lumbosacral spine FE models subjected to the daily cyclic loading provided an enhanced possibility to include the effect of damping characterization in the achieved results. For this purpose, disc height loss and fluid loss were chosen in the current comparative investigation as two critical indicators of the initiation of IVD denaturation which may lead to mild/severe IVD degeneration in a couple of years. Applying repetitive compressive loading to pre-op models results in an approximately uniform disc height reduction which is consistent with previous studies in the literature (Tyrrell et al., 1985; Lai et al., 2008). The induced modification regarding the interbody fusion in post-op models alters the load sharing and motion patterns by increasing the stiffness of the instrumented segment which leads to increased IVD height loss and fluid loss. Although no statistical differences were calculated for pre-op FE results between the two groups, the post-op FE results demonstrated that both IVD height loss and fluid loss significantly increased for the patients with ASD. Hence, the results of this study highlighted the effect of geometrical parameters (which may refer to the anatomical conditions or the induced modifications regarding surgical techniques) on the time-dependent response of lumbar spine biomechanics. The variations in IVD height loss and fluid loss may perhaps indicate initiation of ASD as it is confirmed for more than half of patients who underwent rigid-fusion surgery in previous clinical investigations (Miyakoshi et al., 2000; Ishihara et al., 2001). The alteration of fluid-solid interaction during the cyclic loading possibly changes the stress/strain distribution in the AF matrix. Increasing the disc height loss and fluid loss decreases the contribution of the fluid resistance in IVD which leads to an increase of stress in the AF matrix and collagen fibers. This is the reason that no significant differences were calculated for IDP between the two groups in static loading but the stress/strain increased after 16 h of cyclic loading. Analogous biomechanical response patterns were calculated for the stress and fiber strain in the disc AF matrix region post-fusion. Increased axial stress was significant in both groups when comparing the pre-op and post-op results for the sagittal plane. However, the ASD group showed significantly higher stress for both flexion and extension movement. An increased amount of stress in the AF matrix refers to decreasing the effect of damping resistive interstitial water in total IVD bulk stiffness (Schmidt et al., 2010; Galbusera et al., 2011a). Hence, it can be related to a higher rate of fluid loss rate after repetitive daily loading. On the other hand, rigid fusion surgery can alter the load sharing by removing the load path from the anterior region (vertebral bodies and IVDs) and shifting it to the posterior fixation system which significantly affects the adjacent IVD loading conditions. In addition, the increased fiber strain in the AF region was observed in upper adjacent levels between the two groups for both the pre-op and post-op models which clearly highlights that this geometrically patient-specific FE modeling can approximately differentiate the non-ASD patients from the patients with ASD.

Some limitations of this work should be acknowledged. We used a geometrically personalized modeling algorithm which means that this methodology is only sensitive to anatomical conditions and the same mechanical properties were assumed for the different components of the lumbosacral spine FE models for both groups. As there is no feasible algorithm to extract the mechanical properties of different tissues from X-ray images and it is very difficult to develop optimization methodology for this purpose, the observed variations in motion patterns and load sharing for different patients are only based on different geometrical conditions in this study. In addition, the main contribution of this study is to investigate if the anatomical parameters alter the biomechanical responses of adjacent segments following lumbar fusion surgery. Hence, this simplification might be tolerated to make this study feasible. Another limitation is neglecting the effect of active muscle force in this geometrically personalized osseoligamentous FE modeling technique. Although the follower load approach (Patwardhan et al., 1999; Shirazi-Adl and Parnianpour, 2000; Dreischarf et al., 2014) was considered in this study to mimic the passive response of muscles and upper body weight, there is a lack of muscle force reaction which may alter the biomechanical response specially for possible post-surgical muscle damages. To augment the personalized muscle forces to the model, new sets of clinical measurements before and after surgery are needed which can be planned in our future works.

## 5 Conclusion

This study used a validated geometrically personalized FE modeling algorithm which has the potential to be used in clinics to analyze the biomechanical response of adjacent segments post posterior lumbar fusion surgery. Evaluating the biomechanical response of pre-op and post-op modeling in the non-ASD versus ASD groups showed that the geometrical differences among patients (such as vertebral geometries, IVD height, lumbar lordosis angle, interbody cage size) cause significant variations in the estimated mechanical response. In addition, significant differences between estimated disc height loss, fluid loss, stress, and strain in adjacent levels for aforementioned patient groups can predict the increased risk of pathological development of ASD, which is consistent with our long-term clinical observation in this study. In conclusion, the results of the current study highlighted the effect of geometrical parameters (which may refer to the anatomical conditions or the induced modifications regarding surgical techniques) on the time-dependent response of lumbar spine biomechanics. Henceforward, performing the simulations based on geometrical personalized FE models which include the patient’s anatomical parameters can be used as a surgical planning tool in clinical settings to minimize further long-term complications. It may provide clinicians with a valuable pre-planning tool to make informed pre-op and post-op decisions.

## Data Availability

The raw data supporting the conclusion of this article will be made available by the authors upon request.
